# Labetalol infusion for refractory hypertension causing severe hypotension and bradycardia: an issue of patient safety

**DOI:** 10.1186/1754-9493-2-13

**Published:** 2008-05-27

**Authors:** Samir Fahed, Daniel F Grum, Thomas J Papadimos

**Affiliations:** 1 Department of Anesthesiology, University of Toledo College of Medicine, 3000 Arlington Avenue, Toledo 43614, USA

## Abstract

Incremental doses of intravenous labetalol are safe and effective and, at times, such therapy may need to be augmented by a continuous infusion of labetalol to control severe hypertension. Continuous infusions of labetalol may exceed the recommended maximum daily dose of 300 mg on occasion. We report a case in which hypertension occurring after an abdominal aortic aneurysm repair, initially responsive to intermittent intravenous beta-blockade, became resistant to this therapy leading to the choice of an intravenous labetalol infusion as the therapeutic option. The labetalol infusion resulted in a profound cardiovascular compromise in this postoperative critically ill patient. While infusions of labetalol have successfully been used, prolonged administration in the intensive care unit requires vigilance and the establishment of a therapeutic rationale/policy for interventions, such as the ready availability of glucagon, β-agonists, phosphodiesterase inhibitors, insulin, and vasopressin when severe cardiovascular depression occurs.

## Background

Labetalol is a non-selective β-adrenergic receptor antagonist, and a post-synaptic α-adrenergic receptor antagonist. It is used in the treatment of essential hypertension, renal hypertension, hypertension of pregnancy, pheochromocytoma, and hypertensive crises. It can be administered orally or intravenously. The β/α ratio of antagonism is 7:1 after intravenous administration (a 3:1 ratio exists after oral administration). The drug is lipid-soluble, has a 25% bioavailability, is devoid of active metabolites, and has a half-life of approximately 5.5 hours. Labetalol decreases blood pressure with a limited effect on cardiac output and heart rate at recommended dosages. Its side effects include postural hypotension/dizziness, fatigue, headache, rashes, impotence, urinary retention, gastrointestinal problems, asthma, Raynaud's phenomenon, and heart failure [1].

Incremental doses of intravenous labetalol have been demonstrated to be safe and effective [2,3]. A continuous infusion of up to 2 mg/min, or intermittent intravenous (IV) injections of 40 mg or 80 mg following an initial injection have been recommended, to a maximum of 300 mg [2-4]. Long term continuous infusions of labetalol that have exceeded the 300 mg maximum recommendation by the manufacturer have successfully been used to control severe hypertension in medical and trauma patients [5,6]. However, profound hypotension has also been associated with an infusion dose that nears or exceeds the maximum recommended [7].

We report a case of labetalol infusion overdose in which profound hypotension and bradycardia occurred in a hypertensive patient after abdominal aortic aneurysm repair. The patient received an infusion that exceeded the manufacturer's recommended cumulative dosage and was rescued with IV glucagon.

## Case presentation

A 75 year old 61 kg white female was admitted to the surgical intensive care (SICU) unit after an elective abdominal aortic aneurysm repair. Past medical history included hypertension, renal cell carcinoma, left breast cancer, and peripheral vascular disease. Past surgical history included tonsillectomy, hysterectomy, cholecystectomy, left nephrectomy, left adrenalectomy, and left breast lumpectomy. Her home medications included nitroglycerin, verapamil, and furosemide. She claimed allergies to sulfa, ciprofloxacin, fexofenadine, codeine, cortisone, phenytoin, fluconazole, metoclopramide, penicillin, cisapride, erythromycin, and sertraline.

**Figure 1:**
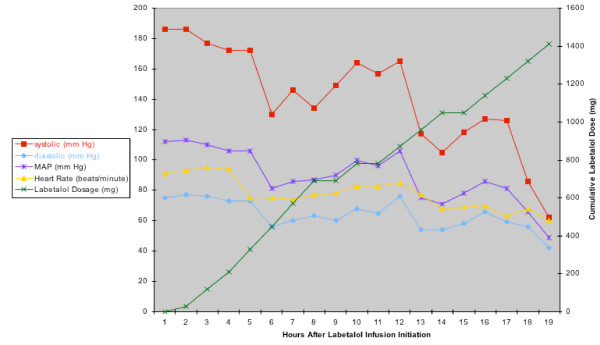
The vital signs and the cumulative labetalol dose over time. MAP = mean arterial pressure; mg = milligrams; and mm Hg = millimeters of mercury.

Intraoperatively her blood pressure was controlled with intravenous nitroglycerin, sodium nitroprusside, and metoprolol, and she arrived in the SICU hemodynamically stable. Her SICU stay was prolonged by a ventilator associated pneumonia and renal insufficiency. For the first twelve postoperative days her hypertension was controlled with intermittent labetalol and a nitroglycerin infusion. On postoperative days 13 through 20 her hypertension required only intermittent IV metoprolol. On postoperative day 21 she developed hypertension that did not respond to intermittent beta blockade (systolic blood pressures of 160–202 mm Hg) and the decision was made to start the patient on a labetalol infusion at 0.5 mg/min. The infusion varied between 0.5 mg/min and 2.0 mg/min. After 16 hours of the infusion her blood pressure dropped to 60/40 mm Hg with a heart rate of 58 beats per minute and a central venous pressure (CVP) of 5 cm H2O. The patient had received 1637 mg of labetalol over 16 hours (102.3 mg/hr, see figure 1). Beta-blocker overdose was suspected and glucagon was ordered, but it was not immediately available. The patient was given one liter of 0.9% NaCl rapidly, ephedrine 5 mg IV twice, and atropine 0.5 mg IV without result. A dopamine infusion of 10 mcg/min was also ineffective. Two doses of 10 mcg of epinephrine IV raised the systolic blood pressure to 70 mm Hg and the heart rate to 65 beats per minute. An epinephrine infusion was then started at 0.05 mcg/kg/min. The glucagon arrived 10 minutes after being ordered and 3.5 mg IV (.05 mg/kg) was given over 3 minutes. The patient's blood pressure promptly recovered to 94/47 mm Hg, heart rate to 73/min, and a CVP of 14 cm H2O. The glucagon bolus was followed by an infusion of glucagon 1 mg/hr for 27.5 hours (approximately 5 half-lives). After the glucagon bolus the patient's blood sugar transiently reached 255 mg/dl. This was corrected with one dose of intravenous insulin and, thereafter, the glucose remained less than 150 mg/dl. The epinephrine infusion was gradually discontinued over 3 hours.

During the acute event the patient's breathing was controlled in a synchronized intermittent mandatory ventilation mode. The FiO2 was increased from 40% to 100% FiO2 with a resultant ABG of pH 7.45, PaCO2 36 mm Hg, PaO2 312 mm Hg, and HCO3 25 meq/L. The patient's cardiac enzymes were normal and the transthoracic echocardiogram demonstrated normal systolic and diastolic function in the face of left ventricular hypertrophy and moderate mitral regurgitation (which were pre-existing). The electrocardiogram was normal and the chest roentgenogram showed no changes from the morning film.

On post-operative day 24 she received a tracheostomy and on hospital day 45 (post-operative day 44) she was discharged to a long-term facility where she expired 6 months later.

## Discussion

Labetalol infusion rates of 0.5–2 mg/minute have been used without incident to achieve blood pressure control [7-10], including long-term continuous infusions of 20–30 mg/hour IV for 5 days [4] and 120–180 mg/hour for 14 days [5] without incident. However, severe refractory hypotension with labetalol may occur, leading to death or severe cardiac dysfunction [11,12]. The maximum cumulative dose of labetalol used in U.S. clinical trials was 300 mg IV [2-4]. This dosing recommendation may easily be exceeded with a five-hour infusion. The blocking of β-receptors prevents the conversion of adenosine triphosphate to cyclic adenosine monophosphate by the responsible G protein, thereby reducing the amount of cytosolic calcium available for muscular contraction [13]. Besides a reduction in cytosolic calcium, fluctuations in glucose levels are possible with β-blocker overdose, but are rare [14,15]. Glucagon is normally secreted by the pancreas in response to hypoglycemia, causing the liver to convert stored glycogen into glucose. Glucagon-treated patients may present with the side effects of nausea, vomiting, hypokalemia, and hyperglycemia [16]. As indicated above, our patient had a transient hyperglycemia with the glucagon bolus, but she did not demonstrate nausea, vomiting, or hypokalemia.

For profound β-blocker blockade 0.05–.15 mg/kg of glucagon is administered IV and then followed by 1–5 mg/hr continuous infusion until symptoms subside (this may take as much as 5 labetalol half-lives) [8]. Severe hypotension may occur at the time of administration [11,17], or several hours after discontinuation [18,19]. A labetalol infusion leading to β-blocker toxicity has been previously reported once. It occurred in a patient with a subarachnoid hemorrhage and multiple cerebral aneurysms who required hypertension control [6]. The fact that labetalol-induced hypotension may be refractory and can lead to cardiovascular compromise and death is an issue of patient safety [11,12].

Glucagon has been previously used as a rescue measure in β-blocker overdose [20,21], as occurred in this case, but evidence of the use of glucagon as the sole rescue drug is limited [21,22]. In this report, the successful use of glucagon was supported by the use of other drugs. In addition to glucagon, intravenous epinephrine [13], phosphodiesterase inhibitors [18,21,23], insulin [24], and vasopressin [6,25] have also been effective. The mechanisms of action for these drugs are as follow: (1) epinephrine binds to β-receptors that are not bound by the β-blocker, thus activating adenyl cyclase through the G_s _protein, resulting in the release of calcium via the adenosine triphosphate – adenosine monophosphate (AMP) – protein kinase A pathway [13]; (2) glucagon acts directly on the G_s _protein foregoing the β-receptors entirely [13]; (3) phosphodiesterase inhibitors prevent the degradation of cyclic AMP [13]; (4) insulin allows the uptake of carbohydrates (energy), it is anti-inflammatory, and is associated with potassium influx that results in prolonged repolarization allowing calcium channels to remain open for a longer time [13]; and (5) vasopressin works through the V1A receptor where its activity is mediated by G proteins which stimulate a phosphatidylinositol-calcium secondary messenger system [26]. Furthermore, Jiraj et al have hypothesized that some patients may have significant pharmacologic/physiologic sensitivities to the α1 blocking and/or β2 agonist properties of labetalol, therefore vasopressin may be effective through the V1 receptor [6,25]. Profound hypotension in the face of vasopressors may indicate a disabled vascular response, or vasoplegia, which may be due to genetic variability [27].

At our institution vasopressin, phosphodiesterase inhibitors, insulin and vasopressin are more readily available than glucagon. However, except for the use of epinephrine, we did not consider these medications during this clinical event. Additionally, interventions other than a labetalol drip could have been entertained for the control of blood pressure. According to the Vascular Biology Working Group additional options such as a nicardipine infusion, or sodium nitroprusside infusion (with or without β-blockers or angiotensin converting enzyme inhibitors) should be considered in hypertensive urgencies and emergencies [28].

Figure 1 demonstrates that there was a decrease in systolic, diastolic, and mean blood pressures 1–2 hours beforehand, and, therefore, the more serious drop in blood pressure may have been preventable. Our report is only the second case that we can identify in the literature in which a labetalol infusion has been documented to cause a condition of cardiovascular collapse secondary to β-blocker toxicity.

## Conclusion

The medical literature indicates that labetalol is relatively safe to use. However, vigilance is required when it is administered in the form of an intravenous infusion because of the frequency with which the recommended intravenous dose may be exceeded with its prolonged use. We recommend that surgical intensive care units using labetalol infusions to control refractory hypertension have glucagon, epinephrine, insulin, phosphodiesterase inhibitors, and vasopressin readily available for administration.

## Competing interests

The authors declare that they have no competing interests.

## Authors' contributions

SF and TJP cared for the patient, SF, DFG, and TJP all contributed equally to the manuscript.

## Consent

consent was obtained from the patient's husband for publication.
